# The Molecular Basis for the Broad Substrate Specificity of Human Sulfotransferase 1A1

**DOI:** 10.1371/journal.pone.0026794

**Published:** 2011-11-01

**Authors:** Ilana Berger, Chen Guttman, Dotan Amar, Raz Zarivach, Amir Aharoni

**Affiliations:** 1 Department of Life Sciences, Ben-Gurion University of the Negev, Be'er Sheva, Israel; 2 National Institute for Biotechnology in the Negev, Ben-Gurion University of the Negev, Be'er Sheva, Israel; University College Dublin, Ireland

## Abstract

Cytosolic sulfotransferases (SULTs) are mammalian enzymes that detoxify a wide variety of chemicals through the addition of a sulfate group. Despite extensive research, the molecular basis for the broad specificity of SULTs is still not understood. Here, structural, protein engineering and kinetic approaches were employed to obtain deep understanding of the molecular basis for the broad specificity, catalytic activity and substrate inhibition of SULT1A1. We have determined five new structures of SULT1A1 in complex with different acceptors, and utilized a directed evolution approach to generate SULT1A1 mutants with enhanced thermostability and increased catalytic activity. We found that active site plasticity enables binding of different acceptors and identified dramatic structural changes in the SULT1A1 active site leading to the binding of a second acceptor molecule in a conserved yet non-productive manner. Our combined approach highlights the dominant role of SULT1A1 structural flexibility in controlling the specificity and activity of this enzyme.

## Introduction

The cytosolic sulfotransferase (SULTs) family catalyzes the transfer of a sulfate group from the universal 3′-phosphoadenosine 5′-phosphosulfate (PAPS) donor to a wide variety of acceptor molecules bearing a hydroxyl or an amine group [1], [2], [3]. Sulfonation results in inactivation of the majority of acceptors, including neurotransmitters, steroid hormones and drugs, thus modulating their biological activity and rendering the product more soluble and readily excretable. Sulfonation thus provides a line of defense by enabling the detoxification of a variety of chemicals. In some cases, however, sulfonation results in the metabolic activation of carcinogens and mutagens, while natural polymorphism in SULT genes has been shown to be associated with an increased risk for cancer [4].

In humans, 13 different SULT genes have been identified and can be divided into four families, including SULT1, SULT2, SULT4 and SULT6 [5], [6]. Of these, the SULT1 family is the largest and is responsible for the sulfonation of phenol, thyroid and steroid hormones, as well as a variety of xenobiotics and known drugs [7]. The SULT1 family can be further divided into an additional 5 sub-families, each displaying distinct substrate preference and overlapping substrate specificity. For example, SULT1A1 [8] displays substrate preference for small phenolic compounds, while SULT1E1 shows a preference for estrogen acceptors [9]. Despite differences in substrate preference, a considerable degree of structural homology exists between the SULTs, highlighting the difficulties in understanding the molecular basis for the broad specificity of these enzymes [7].

A prevalent phenomenon in sulfotransferase activity is partial inhibition at high substrate concentrations. The X-ray structure of SULT1A1 in complex with *p*-nitrophenol (*p*NP) revealed two *p*NP molecules bound to the enzyme active site, suggesting that substrate inhibition is due to a decreased rate of catalysis when both *p*NP molecules are bound [10]. However, to understand the structural changes leading to binding of a second acceptor molecule to SULT1A1 in a non-catalytic mode, much more detailed structural data is needed.

In this study, we examined the molecular basis for the broad specificity and substrate inhibition of SULT1A1 using structural, protein engineering and kinetic approaches. We determined five new X-ray structures of SULT1A1, including that of SULT1A1 in complex with the 3′-phosphoadenosine 5′-phosphate (PAP) donor product and 3-cyano-7-hydroxycumarin (3CyC) or 2-naphthol (2NAP) acceptors. These structures demonstrate the high plasticity of the SULT1A1 active site in accommodating different types of acceptor molecule. Our SULT1A1 structures in complex with one or two 3CyC molecules provide new insights into the structural basis of SULT1A1 substrate inhibition. Comparison between the two structures shows a dramatic movement of Phe247 allowing the exposure of a conserved binding site for the second 3CyC molecule. Directed evolution of SULT1A1 for enhanced thermostability and catalytic activity allowed the identification of residues found on the protein surface and in the vicinity of SULT1A1 active site, respectively, that play crucial roles in controlling these SULT1A1 properties. Finally, we determined the structure of the SULT1A1 D249G mutant that confers increased activity towards *p*NP and 3CyC, to highlight the importance of structural flexibility in tuning SULT1A1 transfer activity.

## Results

**Figure 1:**
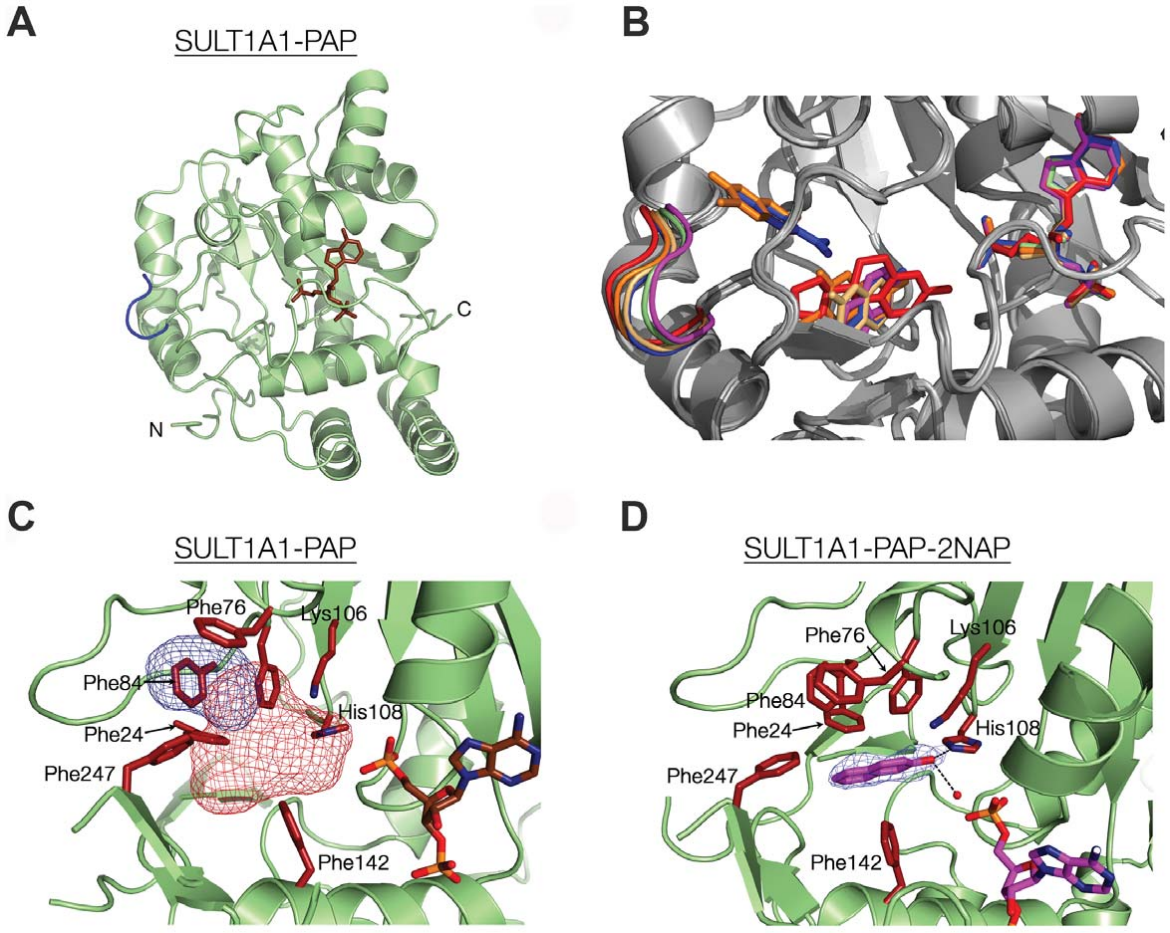
The overall structure of SULT1A1-PAP and flexibility of the SULT1A1 binding pocket. (A) View of SULT1A1-PAP with the PAP molecule colored in brown and the gating loop (residues 86–90) colored in blue. (B) Structural flexibility of SULT1A1 is demonstrated by the overlapping of the donor and acceptor binding pockets of all newly and previously determined SULT1A1 structures (see text for details). The colors of the gating loop, the donor and the acceptors are as follows: SULT1A1-PAP-2NAP-pink, SULT1A1-PAP-green, SULT1A1-PAP-3CyC2-orange, SULT1A1-PAP-*p*NP-blue (PDB code 1LS6), SULT1A1-PAP-E2-red (PDB code 2D06) (C) SULT1A1 in complex with PAP shows the formation of a SULT1A1 empty acceptor binding site comprising pocket-1 and pocket-2, depicted as red and blue mesh blobs, respectively. (D) The structure of SULT1A1-PAP-2NAP. The 2NAP acceptor and PAP donor are colored in pink and the 2NAP is outlined with a *F_O_-F_C_* electron omit map contoured at 2.5ó. Key residues in panels **C** and **D** are colored red.

To obtain detailed understanding of the molecular basis for the broad substrate specificity of SULT1A1, we solved four crystal structures of wild type (WT) SULT1A1 in complex with different acceptors. We determined the structure of SULT1A1 in complex with PAP alone (SULT1A1-PAP), with PAP and 3CyC (SULT1A1-PAP-3CyC1 and SULT1A1-PAP-3CyC2) and with PAP and 2NAP (SULT1A1-PAP-2NAP) to resolutions of 2.0–2.7 Å (**Table S1** and ****S2****). Our overall determined structures resemble the typical α/β core fold of SULT1A1, comprising four β-strands framed by two α-helices on either side [7], [9], [10] (**Fig. 1A**). In all determined structures, as well as in previously described SULT1A1 structures, the PAP moiety is located at the same position in a catalytically competent manner with all binding site residues oriented to minimize conformational freedom of the PAP molecule (**Fig. 1B**) [10], [11].

### Acceptor binding site

The SULT1A1-PAP binary complex structure, in the absence of acceptor, reveals a large L-shaped empty binding site proximate to the PAP donor product (**Fig. 1C**). This empty site is very similar to the binding site that is occupied by the different acceptors (**Fig. 1** and below), indicating that PAP binding leads to the pre-formation of the acceptor binding pocket in SULT1A1. The acceptor cavity can be divided into two compartments, referred to as pocket-1 and pocket-2, formed by Phe24, Phe81, Lys106, His108, Val148 and Phe247 (colored red mesh in **Fig. 1C**) and by Phe76, Phe84, Ile89, Tyr240, and Phe247 (colored blue mesh in **Fig. 1C**), respectively. We found that SULT1A1 crystals formed only in the presence of PAP, suggesting that PAP binding leads to dramatic conformational changes in the protein, as observed in the case of the SULT1A3 structure [12], [13].

The refined 3D structure of SULT1A1-PAP-2NAP revealed a single 2NAP molecule within pocket-1, positioned in a catalytically competent manner in which the hydroxyl group is positioned 2.34 Å from the catalytic amine group of His108 and 3.33 Å from Lys106 (see **Fig. 1D**). The position of 2NAP is stabilized by hydrogen bonding with a nearby water molecule, as well as by stacking hydrophobic interactions with pocket-1 hydrophobic residues.

**Figure 2:**
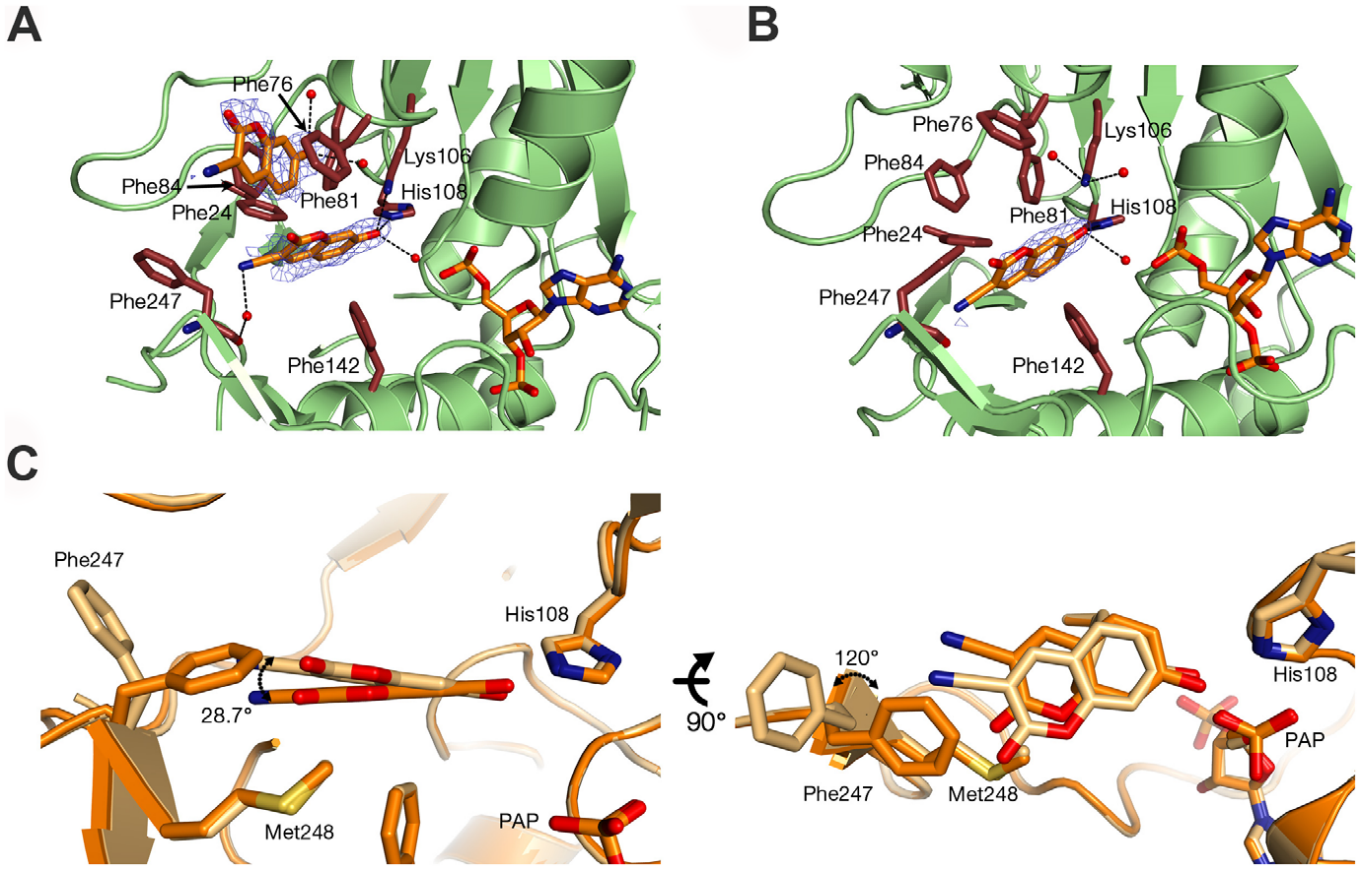
The molecular basis of SULT1A1 3CyC substrate inhibition. Structure of SULT1A1 in complex with PAP and two molecules of 3CyC (SULT1A1-PAP-3CyC2, A) or one molecule of 3CyC (SULT1A1-PAP-3CyC1, B) bound at the acceptor-binding site. Key residues are highlighted in red and the 3CyC molecules are outlined with *F_O_-F_C_* electron omit maps contoured at 2.5ó. (C) Superposition of 3CyC molecules and key residues within the SULT1A1-PAP-3CyC1 and SULT1A1-PAP-3CyC2 structures indicating dramatic movements of the 3CyC cyano group and of Phe247.

We determined SULT1A1 structures in complex with PAP and one or two molecules of the 3CyC acceptor (SULT1A1-PAP-3CyC1 and SULT1A1-PAP-3CyC2, respectively). The SULT1A1-PAP-3CyC2 was probably formed due to drop dehydration leading to increased 3CyC concentration. These structures allowed us to examine the changes undergone by SULT1A1 upon binding of one versus two 3CyC molecules. In the SULT1A1-PAP-3CyC2 structure, the first 3CyC molecule (3CyC1) is located in a catalytically competent position with its first phenol ring fitting in between Phe142 and Phe81 in pocket-1, and its hydroxyl group forming hydrogen bonds with the catalytic His108 and Lys106 residues (**Fig. 2A**). The second 3CyC molecule, 3CyC2, is stacked between Phe76 and Phe84 at a 90° degree planar rotation in relation to 3CyC-1 (**Fig. 2A**). The SULT1A1-PAP-3CyC1 structure, containing only one 3CyC acceptor (**Fig. 2B**), demonstrates similar features to the SULT1A1-PAP-3CyC2 structure, although several important differences exist. The most prominent difference was identified at Phe247, which is flipped 100° towards pocket-1 in a similar manner as seen in the SULT1A1-PAP and SULT1A1-PAP-2NAP structures (**Fig. 2C**). The location of Phe247 leads to a vertical shift in the position of the 3CyC phenol ring by 28.7°. Phe247 is thus partially stabilized by a π-π interaction between the cyanide group of 3CyC and Phe247 and by hydrogen bonding to the Phe247 carbonyl (distance 3.76 Å; **Fig. 2B**). The flexibility of 3CyC-1 in pocket-1, affecting Phe247, can lead to the binding of a second 3CyC molecule to pocket-2, thereby affecting the catalytic efficiency of sulfate transfer (**Fig. 2C**). However, it is possible that 3CyC-2 binding is leading to conformational changes in the SULT1A1 active site affecting Phe247.

### Comparing the different SULT1A1 structures

**Figure 3:**
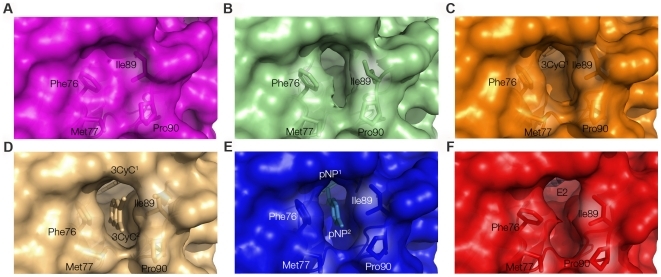
Surface representation of the binding pocket and pore size of the different SULT1A1 structures. Ligands and binding pockets are colored according to the displayed SULT1A1 structure: SULT1A1 in complex with PAP and 2NAP (A, pink), PAP (B, green), PAP and 3CyC1 (C, containing one 3CyC molecule, orange), PAP and 3CyC2 (D, containing two 3CyC molecules, grey), PAP and *p*NP (E, blue) or PAP and E2 (F, red). Key residues are highlighted in stick-form. Newly determined structures are A–D.

Comparing the four newly determined structures of WT SULT1A1 together with the previously determined structures SULT1A1-PAP-*p*NP and SULT1A1-PAP-E2 [10], [11] enabled us to explore conformational changes induced by the binding of diverse acceptors to SULT1A1 (**Fig. 3** and **Table S3**). Identified changes among the different structures were exclusively mapped to those residues shaping pocket-1, representing the catalytically competent binding site (**Fig. 1C**). The most prominent changes were identified at residues 86–90, corresponding to the gating loop (**Fig. 1B**). The gating loop dictates the pocket entrance width and can thus control the entry and evacuation of non-sulfonated and sulfonated molecules, respectively (**Fig. 3**). Comparing the root mean square deviation (RMSD) values of the SULT1A1-PAP structure gating loop with that of SULT1A1-PAP-2NAP, SULT1A1-PAP-3CyC1, SULT1A1-PAP-pNP, SULT1A1-PAP-3CyC2 and SULT1A1-PAP-E2 demonstrated significant differences (1.08, 1.28, 1.4, 1.57 and 2.04 Å, respectively) which correlated with a gradual withdrawal of the loop towards the solvent (**Fig. 1B**). Comparing the dimension properties of the acceptor pockets (i.e., cavity volume, pore area and pore width) among the different structures reveals a general correlation between a withdrawal of the gating loop and an increase in the volume of the cavity (**Fig. 1** and **Table S3**), illustrating the plasticity of the binding site to accommodate diverse acceptors.

Comparing the structures of SULT1A1-PAP-2NAP and SULT1A1-PAP-3CyC1 (**Fig. S1**) reveals that the binding of a 2NAP molecule, which is more hydrophobic than 3CyC [14], leads to inward movement of the gating loop. This, in turn, dramatically decreases the distance between Ile89 and Phe76 (3.84 versus 6.60 Å in SULT1A1-PAP-2NAP and SULT1A1-PAP-3CyC1, respectively), compacting pocket-2 and the entrance to the pore (see **Fig. S1** and **Table S3**). It thus appears that the hydrophobic nature of 2NAP is sufficient to create additional hydrophobic interactions with Phe84 and Phe247 that drive the closure of the binding pocket in such a way that deflects any additional 2NAP molecules from entering the non-catalytic pocket.

### Directed evolution of SULT1A1

To obtain a deeper understanding of the structure-function relationship of SULT1A1, we employed a directed evolution approach to obtain SULT1A1 mutants showing enhanced thermostability and catalytic activity (**Fig. S2**). The directed evolution approach involves two major steps: (i) the generation of genetic diversity in the gene of interest to obtain large mutant libraries and (ii) the selection of these libraries for the desired catalytic or binding activity [15], [16], [17]. As a first step in the evolution of SULT1A1 for novel specificity, we sought to improve SULT thermostability. It was previously shown that highly thermostable enzymes that readily express in *E. coli* serve as an excellent starting point for further directed evolution efforts aiming for enhanced catalytic activity and novel specificity [18]. In addition, a trade-off was observed between thermostability and change in enzyme catalytic activity [18]. Thus, mutations that can alter SULT1A1 catalytic properties can lead to reduced thermostability and expression of active protein in *E. coli*.

### Generation of SULT1A1 gene libraries

An effective approach for generating gene libraries that are highly enriched in thermostable mutants involves the targeted mutagenesis of residues that deviate from the consensus sequence of the family back to the consensus sequence [19]. To identify such residues, 34 mammalian SULT1 sequences, corresponding to 8 SULT1A1 homologues, were aligned. Comparison of the SULT sequences revealed that 13 different positions in human SULT1A1 deviate from the consensus sequence (**Table S4**). These residues were thus targeted by mutagenesis. We incorporated the ‘back-to-consensus’ mutations into SULT1A1 using the ISOR (Incorporation of Synthetic Nucleotide via Gene Reassembly [20]) methodology for partial mutagenesis of the targeted positions. Following library generation, sequencing of 10 random SULT1A1 library variants revealed an average of 6 ‘back-to-consensus’ mutations per gene. Each library variant carried a random, and different, subset of mutated residues, with the entire set being represented in the library (data not shown).

### Screening of the ‘back-to-consensus’ library for enhanced thermostability

**Figure 4:**
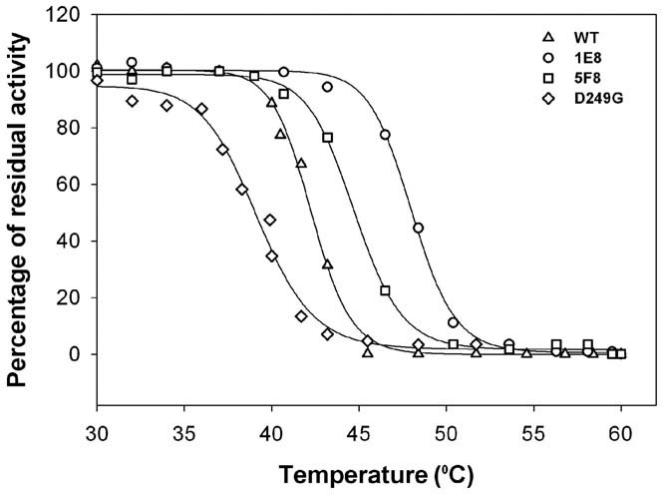
Heat inactivation curves for the WT SULT1A1 protein and the 5C2, 5F8 and D249G SULT1A1 mutants. The 1E8 mutant was isolated after the first round of evolution for increased thermostability and the 5F8 was isolated after the second round of evolution for increased catalytic activity. The decrease in stability of the 5F8 mutant, relative to 1E8, and the D249G mutant, relative to the WT protein, highlight the trade-off between thermostability and the acquisition of new catalytic properties (see text for details).

To isolate SULT1A1 mutants with enhanced thermostability from the ‘back-to-consensus’ library described above, we have developed a simple and rapid high-throughput screening methodology for assessing the transfer of sulfate to 3CyC (**Fig. S2**). This screening assay, performed using *E. coli* crude cell lysates expressing the mutant library clones, follows the quenching of 3CyC fluorescence upon sulfate transfer. We screened ∼600 different mutants from the library for sulfate transfer (SULT) to 3CyC activity by following the time-dependent decrease in the 3CyC fluorescent signal. The top 45 of the SULT1A1 variants exhibiting WT or increased SULT activity were further challenged by heat inactivation at 50°C for 15 min and the residual sulfate transfer activity to 3CyC was measured. These conditions significantly reduced the activity of the WT protein and thus, were optimal for identifying thermostable SULT1A1 mutants. The 10 mutants exhibiting the highest thermostability were sequenced. We found combinations of all of the ‘back-to-consensus’ mutations in the selected SULT1A1 mutants (**Table S5**). This indicates that such mutations are either neutral or beneficial for the catalytic activity and thermostability of the mutants. Selected mutants were over-expressed in *E. coli* cells and purified using Ni-NTA affinity chromatography. Examination of the sulfate transfer activity of these mutants to 3CyC following prolonged incubation at different temperatures revealed an up to 7°C improvement in the heat-inactivation temperature, relative to WT SULT1A1 (**Fig. 4** and **Table 1**). Mapping the ‘back-to-consensus’ mutations on the SULT1A1-PAP crystal structure revealed that many of the mutations are scattered on the outer surface of the protein (**Fig. S3**). Some of the mutations were characterized by a substitution of hydrophobic residues with charged residues (**Table S5**), resulting in reduced hydrophobicity of the surface and promotion of electrostatic interactions with the solvent. However, due to the large number of mutations, it is difficult to dissect the contribution of each mutation to the overall thermostability. Still, it is clear that enhanced thermostability is attained by diverse contributions from the ‘back-to-consensus’ mutations.

**Table 1:** Mutations and heat inactivation temperatures^a^ of newly evolved SULT1A1 variants.

SULT1A1 variants	Mutations	Heat inactivation temperatures^a^
WT	-	42.3±0.1
1E8 (R1)	Q56E, A101S, T117S, Q177K, M223I, V243L	48.1±0.1
1E9 (R1)	L67V, A101S, Q177K, V211L, F222K, F247I	48.3±0.4
5F8 (R2)^b^	Q56E, A101S, T117S, H213R, F222L, V243L, T266N, **L111P, Y240C**	44.7±0.1
5C2 (R2)^b^	Q56E, L67V, A101S, T117S, H213R, F222L, V243I **D249G**	43.3±0.3
D249G^c^	D249G	39.3±0.2

a Heat inactivation temperatures were measured by testing SULT1A1 residual activity following incubation at different temperatures (see **Fig. 4** and Material and Methods).

b Mutations inserted by random mutagenesis in the second round of evolution are highlighted in bold (see text).

c The D249G mutation was generated on the background of the WT protein.

### Screening and isolation of SULT1A1 mutants with enhanced catalytic activity

**Figure 5:**
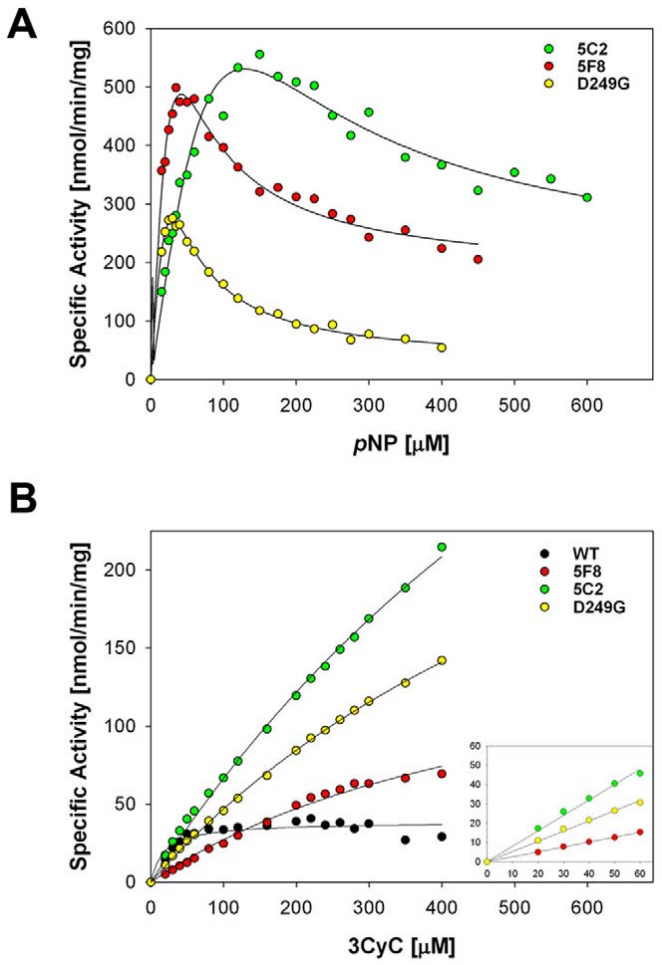
Kinetic analysis of the sulfate transfer activity of the WT SULT1A1 protein and the 5C2, 5F8 and D249G SULT1A1 mutants to pNP (A) and 3CyC (B). Each data point represents the mean of three independent experiments. The lines in A represent fit to data obtained with pNP of equation 1 (see Material and Methods) adopted from ref. 21, taking into account the inhibition seen at high pNP concentrations. The lines in B represent fit to Michaelis-Menten equation of data obtained with 3CyC or linear fit to data obtained at low 3CyC concentrations (inset). The k_cat_/K_M_ parameters derived from the fits are 1208, 466, 149 and 308 for the WT, 5C2, 5F8 and D249G, respectively, presented as sec^−1^ M^−1^.

**Figure 6:**
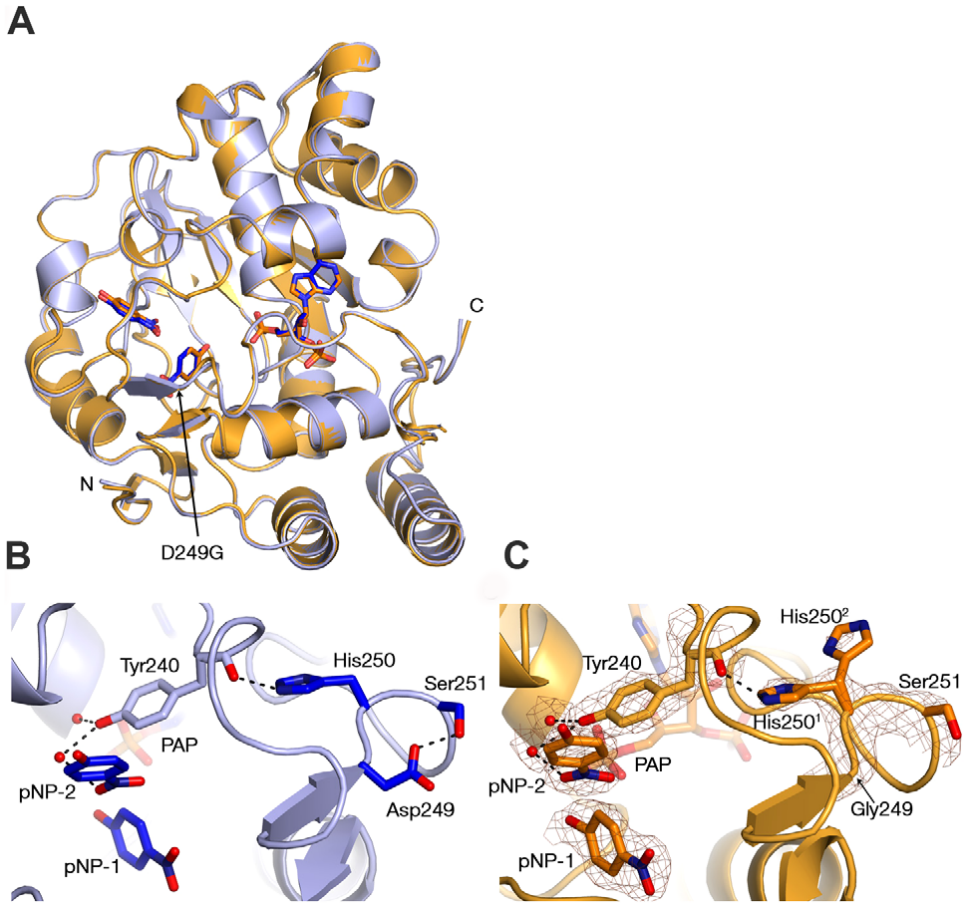
Comparison of the SULT1A1 and SULT1A1-D249G structures in complex with PAP and *p*NP. (A) Structural superposition of SULT1A1 (PDB code 1LS6, light blue) and SULT1A1-D249G mutant (gold). (B–C) View of SULT1A1 (B) and the SULT1A1-D249G mutant (C) ligand pocket and the stabilizing loop containing Asp249 or Gly249 (rotamers are indicated with superscript). SULT1A1-D249G key residues and pNP molecule are outlined with a 2*F_O_-F_C_* electron density map contoured at 1.6ó.

To select for SULT1A1 mutants with increased catalytic activity, we employed genes encoding the 10 most highly thermostable versions SULT1A1 as template for the generation of a random mutant library. Next, we screened ∼700 mutants for SULT-mediated modification of the pNP and 3CyC acceptors at 100 µM and 10 µM, respectively (see Material and Methods for details). Due to the low K_M_ of SULT1A1 for pNP [10], [21] the screening assay should lead to the isolation of mutants with increase in maximal velocity rather than a decrease in K_M_ for this substrate (see below). Using the screening assay, we identified several mutants with substantially increased catalytic activity to these acceptors, including the Y240C and D249G, mutations located in a loop region in the vicinity of the SULT1A1 acceptor-binding site (**Table 1** and **Figs. 5** and **6**). To further characterize the effects of these specific mutations, we generated Y240C and the D249G single mutants on the background of WT SULT1A1 through site-directed mutagenesis. We found that the Y240C mutant is prone to aggregation even at room temperature. As such, this mutant was not further analyzed.

To characterize the thermostability and catalytic activity of the evolved SULT1A1 mutants, we over-expressed the mutants in *E. coli* cells and purified the proteins by affinity chromatography. We found that the mutants displayed lower thermostability, relative to the first generation of highly stable precursor mutants, yet were more stable than the WT protein (**Table 1** and **Fig. 4**). In agreement with these results, the D249G point mutant exhibited lower stability, relative to the WT parent protein (**Fig. 4**). These results highlight the trade-off between the acquisition of increased catalytic activity and thermostability. Next, the catalytic activity of the WT protein and different mutants with the pNP and 3CyC acceptors was measured using steady state kinetics. The kinetic analysis of the evolved SULT1A1 mutants with pNP showed the characteristic increase in SULT1A1 activity at low pNP concentrations and inhibition at high substrate concentrations [10], [21] (**Fig. 5** **,** **Table 2**). The evolved SULT1A1 5F8 and 5C2 mutants (**Table 2**), as well as the D249G mutant, exhibited similar or lower catalytic efficiency (k_s_, **Table 2**) but significantly increase in the maximum velocity (V_p_) relative to the WT enzyme (**Table 2**). Hence, our data together with previous analysis of the WT enzyme using colorimetric and radioactive assays [21], [22] indicate that the mutations in these evolved mutants led to increased catalytic activity at high pNP concentrations but reduced affinity to the first and second pNP molecules occupying the SULT1A1 active site. Due to the low K_M_ of WT SULT1A1 to pNP [21], [22] and the relatively low sensitivity of our non-radioactive assay, we could not monitor the activity of WT SULT1A1 at low pNP concentrations.

**Table 2:** Kinetic parameters of SULT1A1 wild-type and newly evolved 5C2, 5F8 and D249G mutants with pNP and 3CyC.

SULT1A1	pNP			3CyC
	k_s_ a *sec^−1^ M^−1^*	V_p_ a *nmol/min/mg*	V_∞_ a *nmol/min/mg*	k_cat_/K_M_ c *sec^−1^ M^−1^*
WT^b^	12857	92.5	-	1208
5C2	4783±175	441±28	165±28	466
5F8	13591±466	393±18	174±10	149
D249G	10675±233	259±6	26±3	308

a Kinetic parameters for sulfate transfer to pNP were determined by fitting the experimental data to the equation described in ref 21 (see also Material and Methods), taking into account the inhibition observed at high pNP concentrations. The parameter k_s_ is the specificity constant at low pNP concentrations and is equivalent to k_cat_/K_M_ in Michaelis-Menten kinetics. V_p_ is the rate at the peak and V_∞_ is the limiting rate when pNP reaches ∞ (Fig. 5).

b The WT parameters for pNP are adapted from Alcombri et al.[22] the k_cat_/K_M_ is equivalent to k_s_ and V_max_ is equivalent to V_p_.

c The k_cat_/K_M_ values for 3CyC are derived from the fit to Michaelis-Menten equation of data obtained with 3CyC for the WT or linear fit to data obtained at low 3CyC concentrations for the different mutants (Fig. 5).

In contrast, kinetic analysis of the activity towards 3CyC indicated no inhibition at high substrate concentrations for the WT and the mutant proteins. WT SULT1A1 exhibited a much higher K_M_ of ∼20 µM for 3CyC, relative to the pNP, in accordance with the role of SULT1A1 in the sulfonation of small phenolic substrates [8]. The evolved mutants, as well as the D249G point mutant, exhibit higher K_M_ values, relative to the WT protein, and no saturation at concentrations up to 400 µM 3CyC (**Fig. 5**). Overall, the 5C2, 5F8 and D249G mutants exhibit increased sulfate transfer activity to pNP and 3CyC at high substrate concentrations and reduced affinity for these acceptors.

### Structural analysis of SULT1A1 D249G mutant

To obtain deeper understanding of the structural differences between the D249G mutant and the WT protein, we determined the structure of the D249G mutant in complex with PAP and pNP (D249G-PAP-pNP). Comparing the mutant to the previously determined SULT1A1-PAP-pNP structure [10] indicates high structural similarity, with calculated RMSD values of the total atoms of 0.89Å (**Fig. 6A**). However, we found significant local structural changes in the vicinity of the D249G mutation. The substitution of aspartic acid by glycine leads to a loss of hydrogen bonding with Ser251 and possible destabilization of the loop (**Fig. 6**). This destabilization can further disrupt and break the interaction between His250 and the facing carbonyl of Tyr240 by swiveling the His by 100° toward the solvent, as exemplified by the presence of an additional rotamer (**Fig. 6**). Comparison of many SULTs structures shows that the interaction between His250 and the carbonyl of Tyr 240 is conserved in the majority of SULTs, highlighting the importance of this interaction in controlling loop stability (**Fig. S4**). This loss of connection may increase the flexibility of the SULT1A1 binding site, leading to decreased affinity for pNP and 3CyC molecules (**Fig. 6**). It should be noted that the density surrounding the naturally occurring His250 rotamer is stronger than that of the alternative rotamer. We thus assume that in the current crystal packing, this is the ruling conformation that maintains the native position of Tyr240 within the binding pocket (**Fig. 6**). As this phenomenon was not observed in any of the structures of WT SULT1A1 in complex with different acceptors, the importance of Asp249 to the stabilization of the loop and consequently, to the stabilization of Tyr240 within the catalytic pocket, is further supported.

## Discussion

In this work, we employed structural, protein engineering and kinetic approaches to gain detailed understanding of the molecular basis for the broad specificity of SULT1A1. The determination of four new structures of SULT1A1 in complex with the PAP donor and different acceptors enabled us to monitor conformational changes in SULT1A1 upon binding of diverse acceptors. Our results, providing snapshots of SULT1A1 in complex with different acceptors, thus allows us to follow the gradual changes in binding site volume and gating loop movement that occur upon acceptor binding (**Fig. 1** and **Fig. 3**). These results agree with previous structural analysis of different SULT structures determined in complex with different acceptors, demonstrating the high plasticity of the SULT enzyme family [7]. The analysis of the SULT1A1 binary complex with PAP showed that this flexible binding pocket is preformed in the absence of acceptors. Such ‘priming’ was previously shown for various SULTs and suggests ordered binding in SULT1A1, whereby binding of PAPS precedes that of the acceptor [7]. In contrast, it was shown that in the case of SULT2A1, the donor and acceptors can bind independently and that the binding of the acceptor does not necessarily prime the donor binding site [7].

A common characteristic of SULT activity is inhibition at high substrate concentrations. Elegant structural and kinetic characterization of SULT1A1 with pNP indicated two pNP molecules bound at the active site, albeit in different binding modes [10]. It was shown that one pNP molecule is bound in a catalytic mode while the other is bound at a non-catalytic site, probably leading to substrate inhibition [10]. Our determined SULT1A1 structures in complex with one or two 3CyC acceptor molecules indicate that the formation of the binding site for the second 3CyC molecule (3Cyc-2) is probably induced upon binding of the first molecule to the enzyme active site. Comparing the two structures suggests that subtle movements of the 3CyC-1 in the active site leads to substantial movement of Phe247, leading to the exposure of the second 3CyC-binding site (**Fig. 2**). However, it is possible that the binding of the second 3CyC molecule leads to this movement of Phe247. Our kinetic analysis of WT SULT1A1 with 3CyC does not reveal inhibition at 3CyC concentrations of up to 400 µM, indicating that sulfate transfer at these acceptor concentrations takes place in the absence of a second 3CyC molecule bound in a non-productive mode. In contrast, our crystallization efforts performed with SULT1A1 together with 3CyC at high concentration of 1000 µM, yielding crystals of SULT1A1 in complex with one or two 3CyC molecules. These structures, together with the SULT1A1 structure in complex with pNP, indicate a common mechanism for substrate inhibition in SULT1A1 in which a similar binding site is formed at high acceptor concentrations to allow the binding of a second acceptor molecule. Our results are also in correlation with previous studies showing that the SULT1A1 F247L mutation presents substrate inhibition by dopamine, revealing the importance of Phe247 in controlling SULT1A1 substrate inhibition [21]. Interestingly, flipping of the Phe247 was previously identified in SULT1A1 upon binding of estradiol in a non-catalytic orientation [10], [11]. In contrast, it was shown that upon binding of two pNP molecules in SULT1A1 the Phe247 is not flipped [10]. These structures together with the structures described in this study shows that the movement of Phe247 is probably dependent on the acceptor size and is not obligatory to allow the binding of a second acceptor molecule in SULT active site.

To provide further insight into the molecular basis of SULT1A1 catalytic activity, we utilized a directed evolution approach. Accordingly, we first generated highly stable SULT1A1 mutants by insertion of ‘back-to-consensus’ mutations, followed by random mutagenesis to isolate mutants with higher catalytic activity than the WT enzyme. We found that increased SULT1A1 thermostability was probably the result of several stabilizing mutations, rather than being due to one global suppressor, as is the case with the TEM1 β-lactamase M182T mutation [19], [23]. The SULTA1 mutations, mainly localized on the protein surface, conferred up to a 7°C increase in heat inactivation temperature, can buffer the deleterious effects of additional mutations, leading to increased activity. This trade-off between stability and the acquisition of new function has been previously reported for several enzymes [18]. It was previously shown that stabilized P450 and TEM1 mutants exhibit higher evolvability upon the introduction of a large variety of mutations without substantial disruption of the native folding of these proteins [19], [24]. We found that two mutations, namely D249G and Y240C, both located in the vicinity of the active site, can lead to substantial increases in catalytic activity with 3CyC and pNP at high substrate concentrations but lower affinity for these substrates. Structural analysis of the D249G mutant reveals an increase in the local flexibility of this region, resulting in a dramatic movement of His250 that can affect the SULT1A1 active site. The structural conservation of the His250 in many different SULT structures (**Fig. S4**) may highlight the importance of this region for controlling SULT catalytic activity. It is possible that the higher flexibility allows higher substrate turnover but reduces affinity due to changes in the active site. In accordance, electrostatic surface potential analysis of the D249G mutant relative to the WT enzyme indicates a dramatic change in charge distribution around the active site (**Fig. S5**). These changes can lead to reduced affinity for the different acceptors due to possible differences in acceptor binding or effects on the dynamics and flexibility of SULT1A1.

In summary, our work provides new insight into the molecular basis for the broad specificity of SULT1A1 and highlights the importance of structural flexibility for the recognition of a variety of substrates and for controlling SULT1A1 catalytic properties. The use of structural, engineering and kinetic approaches as employed here can be highly beneficial for understanding the molecular basis for the broad specificity of many other liver enzymes that can detoxify a wide variety of substrates, including amidases, monooxygenases, and other transferases.

## Materials and Methods

### Crystallization conditions, diffraction measurements and structural determination

All crystallization conditions and statistics for the five new SULT1A1 crystal structures are provided in **Tables S1** and ****S2****. Grown crystals were mounted in cryoloops at −180°C and collected X-ray diffraction data sets were reduced and scaled using the HKL2000 suite (SULT1A1-PAP and SULT1A1-PAP-2NAP) [25] or iMosflm and Scala (SULT1A1(D249G)-PAP-pNP and SULT1A1-PAP-3CyC) [26], [27]. Phases were obtained by molecular replacement with Phaser [28] using the human SULT1A1 structure (PDB code 1LS6) for our diffraction data [10]. Molecular replacement solutions were followed by rigid body refinement, restrained refinement (Refmac5 software package) [29] and manual building (Coot [30]).

### Least-square overlaps and volume calculation

Structural superposition was performed by the SwissPDB viewer alternate domain fitting function [31]. Ligand pocket volume calculations were established by subtracting the molecular surface of protein complexed with donor and acceptor from the molecular surface of protein complexed with donor alone. The cavity volume of the SULT1A1-PAP-2NAP structure was directly calculated by the software. The cavity volume of the SULT1A1-PAP structure was calculated by the inclusion of two pNP molecules according to their location by superimposing 1LS6 on the structure of SULT1A1-PAP. The cavity volume of the total SULT1A1-PAP-3CyC2 structure was calculated by inclusion of molecule 3CyC2 from the SULT1A1-PAP-3CyC1 structure.

### Calculation of electrostatic potential and binding pocket cavity

Electrostatic calculations were performed by PyMOL [32] using the Adaptive Poisson-Boltzmann Solver (APBS) plug-in [33]. The ligand binding pockets of the hSULT1A1-PAP structure were calculated with the Hollow program [34], setting the solvent-accessible surface around the binding pocket to a probe radius of 1.4 Å.

### Plasmids and bacterial strains

The *E. coli* DH5α and Clooni (Lucigen) strains were used for cloning. The *E. coli* BL21 (DE3) strain was used for protein expression and purification. Human cDNA was used as template for amplification of the human SULT1A1 gene. The amplified gene was cloned into bacterial vector pET32tr (a version of pET32 (Novagen) with truncated thioredoxin) using the NdeI and XhoI sites.

### Synthetic shuffling

SULT1A1 amino acid sequences from eight mammalian species were aligned using Muscle software (EMBL-EBI). Oligo-nucleotides used for synthetic shuffling were 31–33 bases long and contained the ‘back-to-consensus’ single mutation flanked by 15 bases complementary to the SULT1A1 gene at both ends. The *SULT1A1* gene was PCR amplified and 6–10 µg of the PCR products were digested by DNaseI. Fragments (approximate size, 80–120 bp) were extracted and purified. To incorporate the oligonucleotides, the purified fragments were mixed with 5–10 nM of the oligonucleotides and subjected to assembly PCR. The products of the assembly PCR were directly used as template for the amplification of the full length SULT1A1 library containing the spiked oligonucleotides, digested with NdeI and XhoI and cloned into the pET32tr vector as described above.

### Random mutagenesis

Target SULT1A1 sequences were randomly mutagenized by PCR performed with mutagenic dNTP analogs [35]. Mutagenesis stringency was controlled by the number of PCR cycles performed and dNTPs analogs concentration [35].

### Library expression and screening

Single *E. coli* BL21 (DE3) colonies transformed with library or control plasmids were picked to inoculate 600 µl LB media containing 100 µg/ml ampicillin in 96 deep-well plates (Nunc). The plates were incubated with shaking at 37°C overnight. The cells were diluted 1∶50 in fresh selective LB media, incubated with shaking at 37°C until an OD_600_ of 0.4–0.6 and induced with 0.1 mM of IPTG (Calbiochem). The plates were incubated with shaking at 30°C for an additional 5 h and centrifuged at 4000 rpm for 15 min. Media was discarded and the cells were resuspended in 150 µl lysis buffer (2.5 mM MgCl_2_, 0.5 mM CaCl_2_, 0.2% Triton X-100, 2 U/µl DNaseI (NEB), 1 mg/ml lysozyme, 20 mM HEPES, pH 7.5). The plates were incubated with shaking at 37°C for 30 min, followed by 15 min of centrifugation at 4000 rpm. The supernatant (120 µl) was transferred to 96 V shaped-well plates (Nunc) and stored at 4°C for analysis (see sulfotransferase activity assay, below).

### Protein expression and purification

Single colonies were picked to inoculate 5 ml of LB media containing ampicillin (100 µl/ml) and were grown for 16 h at 37°C, diluted 1∶100 with fresh selective LB media and induced with 0.1 mM IPTG (Calbiochem) for an additional 18 h at 20°C. Cells were harvested and centrifuged for 10 min at 6000 g, resuspended in 30 ml 20 mM HEPES, pH 7.5, and recentrifuged for 20 min. Following centrifugation, cells were resuspended in 10 ml/g of binding buffer (1 M NaCl, 40 mM imidazole, 1 mg/ml lysozyme, 20 mM HEPES, pH 7.4). Next, the cells were lysed by sonication, centrifuged and the cleared supernatant was loaded on a pre-equilibrated column containing 2 ml Ni-NTA resin (Qiagen). Following incubation, the resin was washed and SULT1A1 was eluted in 1 ml fractions upon addition of elution buffer. Fractions containing SULT1A1 were analyzed by SDS-PAGE, pooled and dialyzed for 16 h against storage buffer (40 mM HEPES, pH 7.5, 7 mM MgCl_2_, 1.5 mM DTT and 20% glycerol) at 4°C. Protein concentration was determined with a BCA protein assay kit (Pierce). Wash and elution buffers were based on 500 mM NaCl, 20 mM HEPES, pH 7.4 and supplemented with imidazole, according to the manufacturer's recommendations.

### Sulfotransferase activity assays

Clear lysates or purified proteins were added to a reaction mix consisting of the pNP or 3CyC acceptors at different concentrations, 1 mM PAPS, 20 mM HEPES, pH 7.5, 7 mM MgCl_2_, and 1.5 mM DTT to a final volume of 200 µl. The decrease in absorbance (405 nm and 408 nm for pNP and 3CyC, respectively) or fluorescence (3CyC, 408 nm excitation and 450 nm emission) was monitored every 15–40 sec, for at least 20 min, using an ELISA plate reader (Infinite-200, Tecan). Screening of the SULT1A1 library for mutants with increased catalytic efficiency or altered specificity was performed using pNP and 3CyC at 10 µM and 100 µM, respectively. Analysis of the kinetic data obtained with pNP was performed according to ref. 21 by fitting the data to the following equation: *v* = (*k*_s_[S]+*V*_∞_[S]^2^/*K_s_* ^2^)/(1+[S]^2^/*K_s_* ^2^) (equation 1) using SigmaPlot software. The parameter k_s_ is the specificity constant at low pNP concentrations and is equivalent to k_cat_/K_M_ in Michaelis-Menten kinetics. V_p_ is the rate at the peak and V_∞_ is the limiting rate when pNP reaches ∞. Analysis of the kinetic data obtained with 3CyC was performed by fitting the data to the Michaelis-Menten equation or to a linear equation. For heat inactivation analysis, the clear lysates or the purified protein were incubated for 15 min at the desired temperature using a DNA Engine Peltier Thermal Cycler (Bio-Rad) and then assayed as described above.

## Supporting Information

Figure S1 **Comparison of SULT1A1 structures in complex with PAP and 2NAP or 3CyC.** Superposition of the gating loop and key residues indicate a closure of the gating loop, leading to a smaller cavity volume (see main text and **Table S3** for details).
(TIF)

Figure S2 **Scheme describing the directed evolution process and the acceptors used for the analysis of SULT1A1 specificity.** (**A**) The directed evolution process for the generation of SULT1A1 mutants with increased thermostability (R1) and specificity (R2). (**B–D**) Acceptors used for the directed evolution process of SULT1A1 were pNP (**B**), 3CyC (**C**).
(TIF)

Figure S3 **Surface representation model of the human SULT1A1 structure in complex with PAP.** The surface mutations identified in thermostable SULT1A1 mutants (Table 1 and Table S3) are highlighted in blue. The model was generated using the Swiss PDB viewer program.
(TIF)

Figure S4 **Comparison of SULT structures highlighting the loop region containing Tyr240, Asp249, Ser251 and His250.** The high extent of overlap between the loop location and residues demonstrates the structural conservation of this region. In the SULT1A1 D249G mutant, His250 flips about 100° degrees towards the solvent, leading to the loss of interaction between His250 and the carbonyl of Tyr240. The structures that are overlapped are: WT human SULT1A1 in complex with PAP (1A1 w.t., light green), the SULT1A1 D249G mutant in complex with PAP and pNP (1A1 mutant, orange), human SULT1C2 in complex with PAP (1C2, magenta, PDB code 2GWH), mouse SULT1D1 in complex with PAP (1D1, blue, PDB code 2ZYT), human SULT1B1 in complex with PAP and resveratol (1B1, dark orange, PDB code 3CKL), human SULT1C3 in complex with PAP (1C3, dark green, PDB code 2HK8), and human SULT1A2 in complex with PAP (1A2, yellow, PDB code 1Z29).
(TIF)

Figure S5 **Electrostatic surface representation of SULT1A1 and SULT1A1-D249G showing a dramatic change in the surface electrostatics of the two proteins.** Changes in the electrostatics of the surface can affect the pNP acceptor binding site that is located in the vicinity of Asp249. The approximate locations of Asp249 or G249 and the pNP2 molecule are highlighted by arrows.
(TIF)

Table S1 Crystallographic statistics of SULT1A1 structures.
(DOC)

Table S2 Crystallization conditions for SULT1A1.
(DOC)

Table S3 Comparison of SULT1A1 pore size and cavity volume.
(DOC)

Table S4 Amino acids in human SULT1A1 that deviate from the family consensus: Comparison to SULT1A1 and SULT1 families.
(DOC)

Table S5 Mutation distribution in SULT1A1 thermostable mutants.
(DOC)
