# Supplementary figures and images for: The Molecular Basis for the Broad Substrate Specificity of Human Sulfotransferase 1A1
Source: PLoS One. 2011 Nov 1;6(11):e26794. doi: 10.1371/journal.pone.0026794 (PMC3206062; doi:10.1371/journal.pone.0026794)

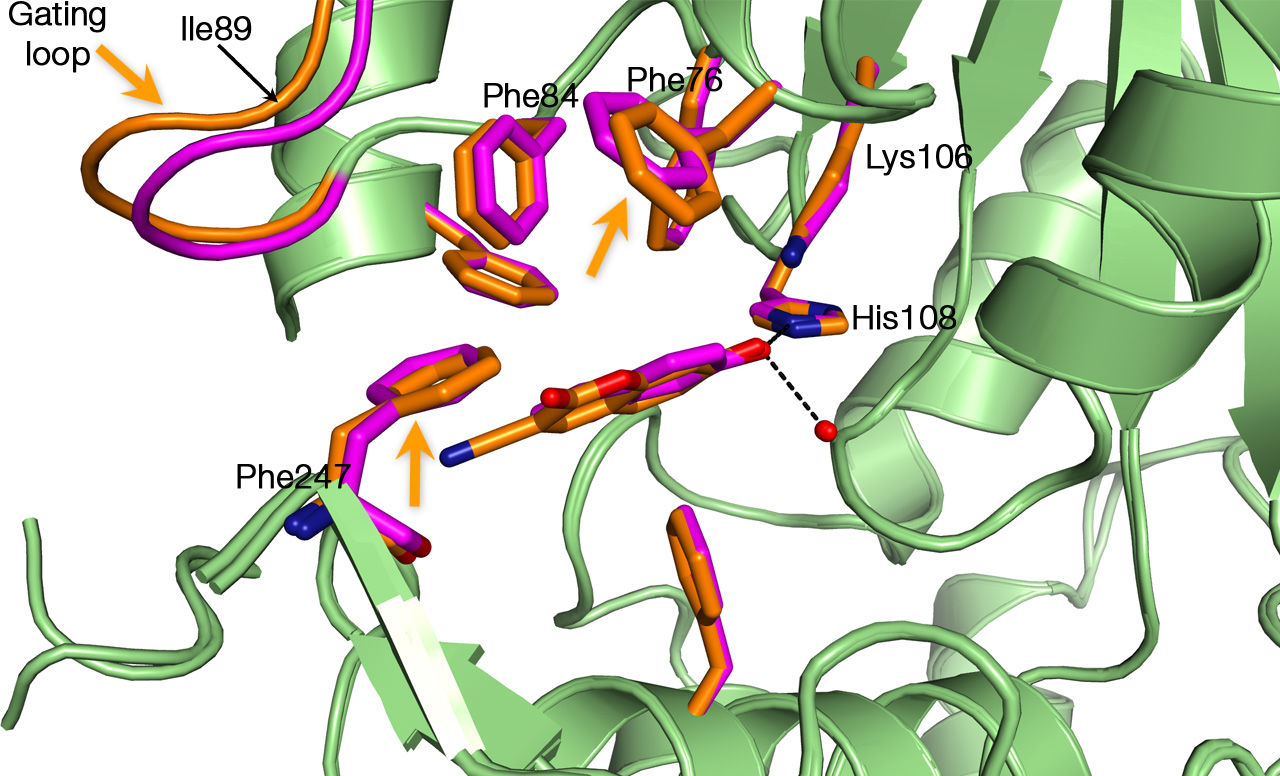

Supplement: Figure S1 — Comparison of SULT1A1 structures in complex with PAP and 2NAP or 3CyC. Superposition of the gating loop and key residues indicate a closure of the gating loop, leading to a smaller cavity volume (see main text and Table S3 for details). (TIF) [file pone.0026794.s001.tif]

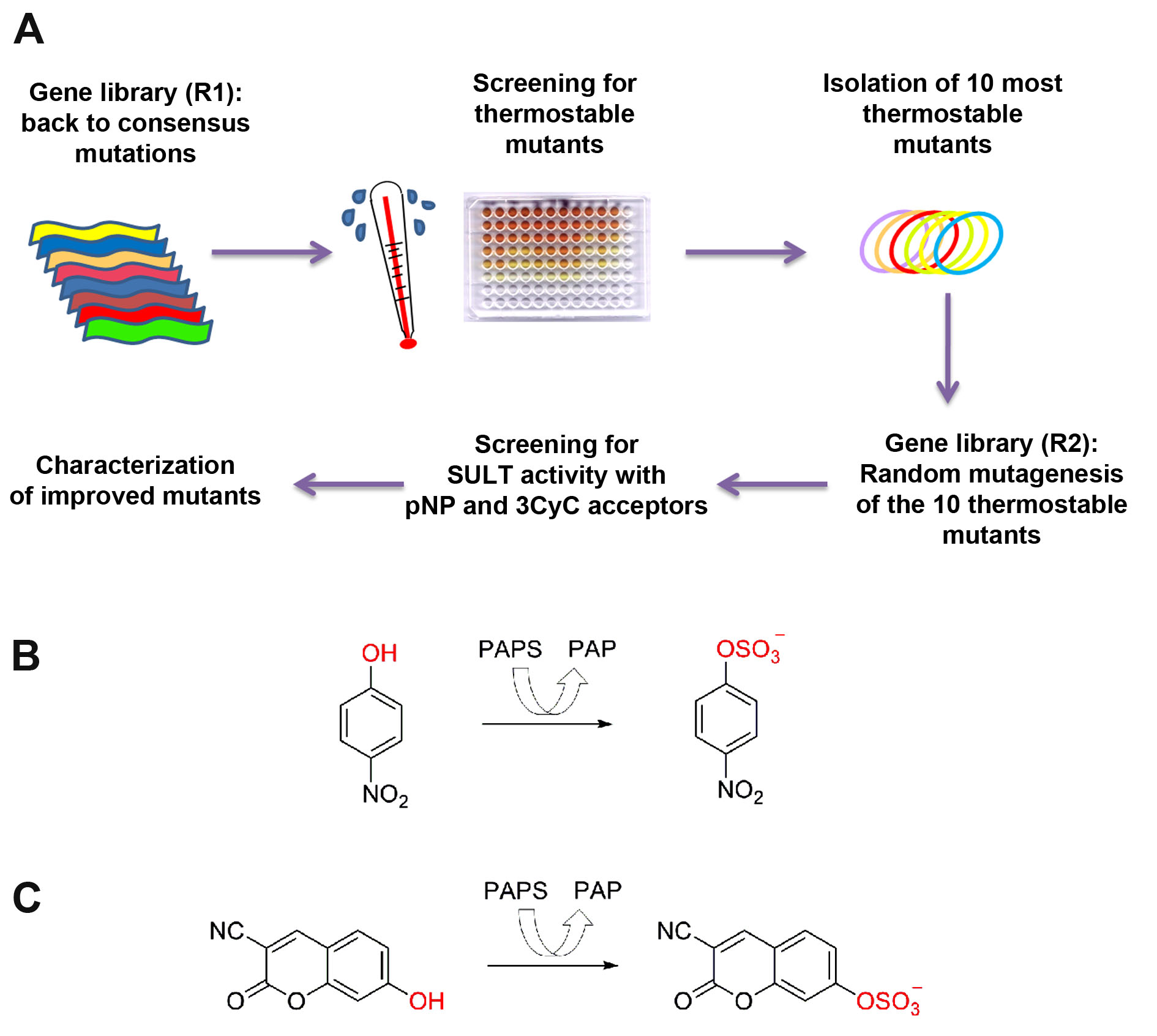

Supplement: Figure S2 — Scheme describing the directed evolution process and the acceptors used for the analysis of SULT1A1 specificity. (A) The directed evolution process for the generation of SULT1A1 mutants with increased thermostability (R1) and specificity (R2). (B–D) Acceptors used for the directed evolution process of SULT1A1 were pNP (B), 3CyC (C). (TIF) [file pone.0026794.s002.tif]

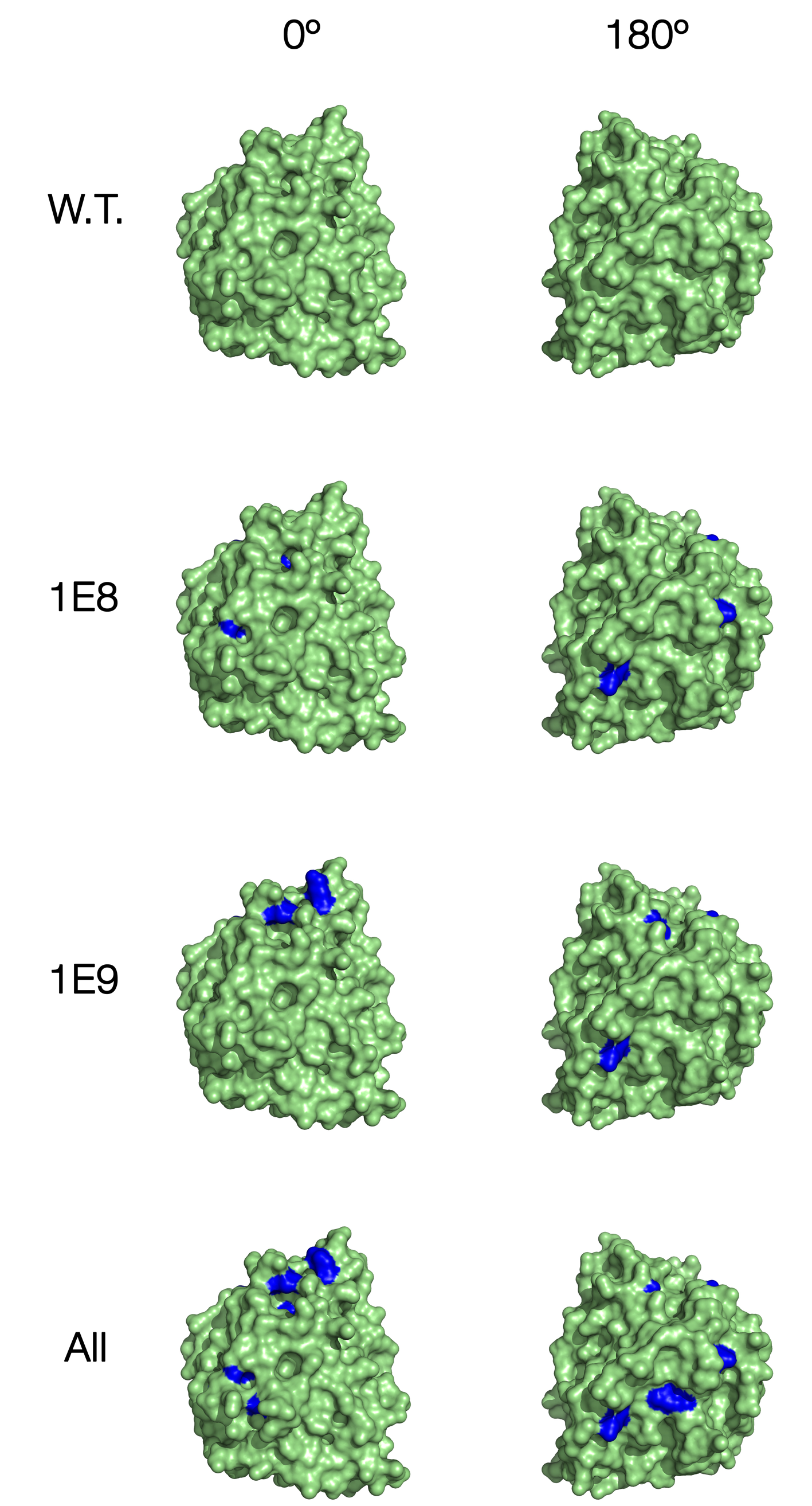

Supplement: Figure S3 — Surface representation model of the human SULT1A1 structure in complex with PAP. The surface mutations identified in thermostable SULT1A1 mutants (Table 1 and Table S3) are highlighted in blue. The model was generated using the Swiss PDB viewer program. (TIF) [file pone.0026794.s003.tif]

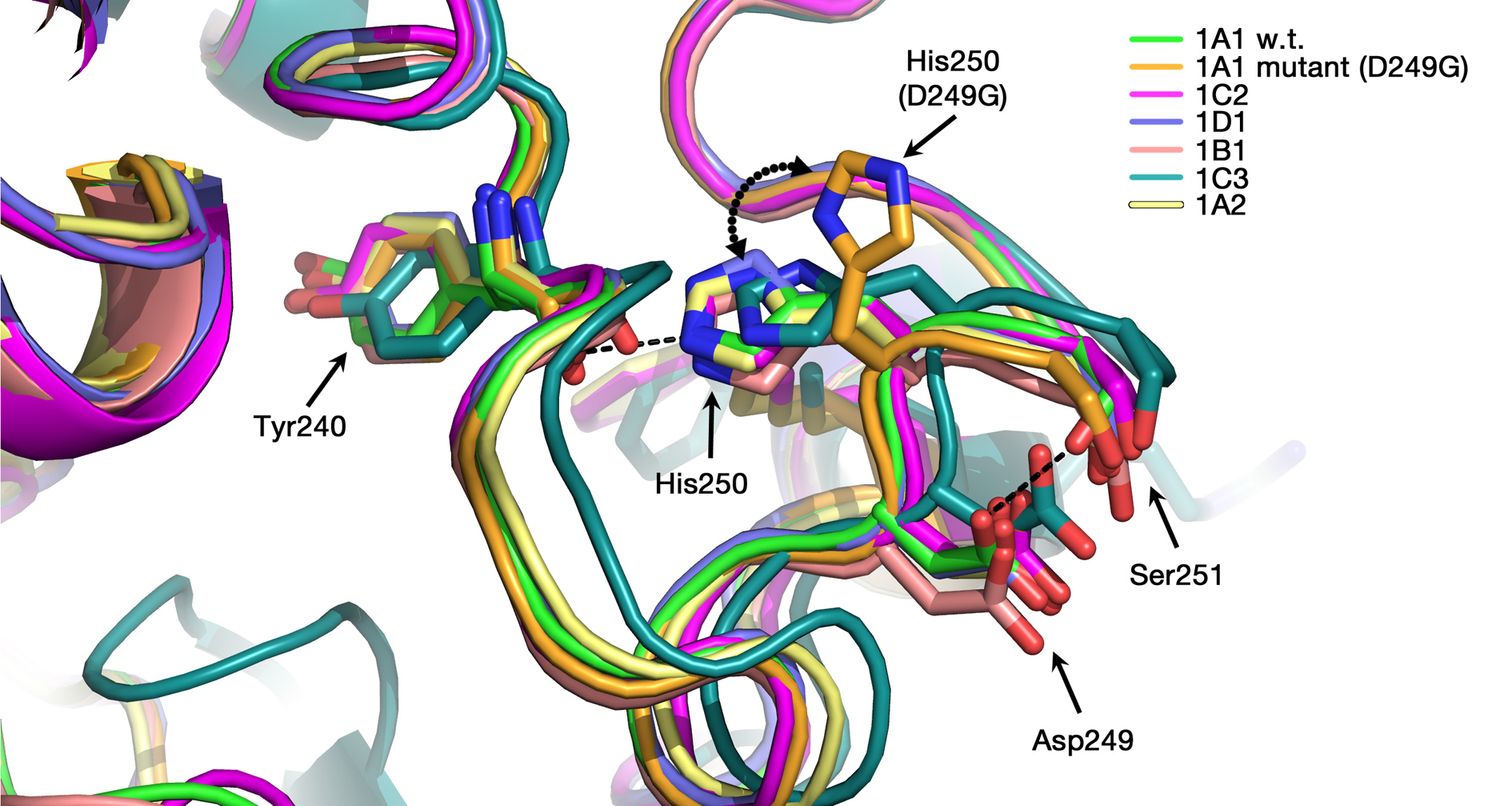

Supplement: Figure S4 — Comparison of SULT structures highlighting the loop region containing Tyr240, Asp249, Ser251 and His250. The high extent of overlap between the loop location and residues demonstrates the structural conservation of this region. In the SULT1A1 D249G mutant, His250 flips about 100° degrees towards the solvent, leading to the loss of interaction between His250 and the carbonyl of Tyr240. The structures that are overlapped are: WT human SULT1A1 in complex with PAP (1A1 w.t., light green), the SULT1A1 D249G mutant in complex with PAP and pNP (1A1 mutant, orange), human SULT1C2 in complex with PAP (1C2, magenta, PDB code 2GWH), mouse SULT1D1 in complex with PAP (1D1, blue, PDB code 2ZYT), human SULT1B1 in complex with PAP and resveratol (1B1, dark orange, PDB code 3CKL), human SULT1C3 in complex with PAP (1C3, dark green, PDB code 2HK8), and human SULT1A2 in complex with PAP (1A2, yellow, PDB code 1Z29). (TIF) [file pone.0026794.s004.tif]

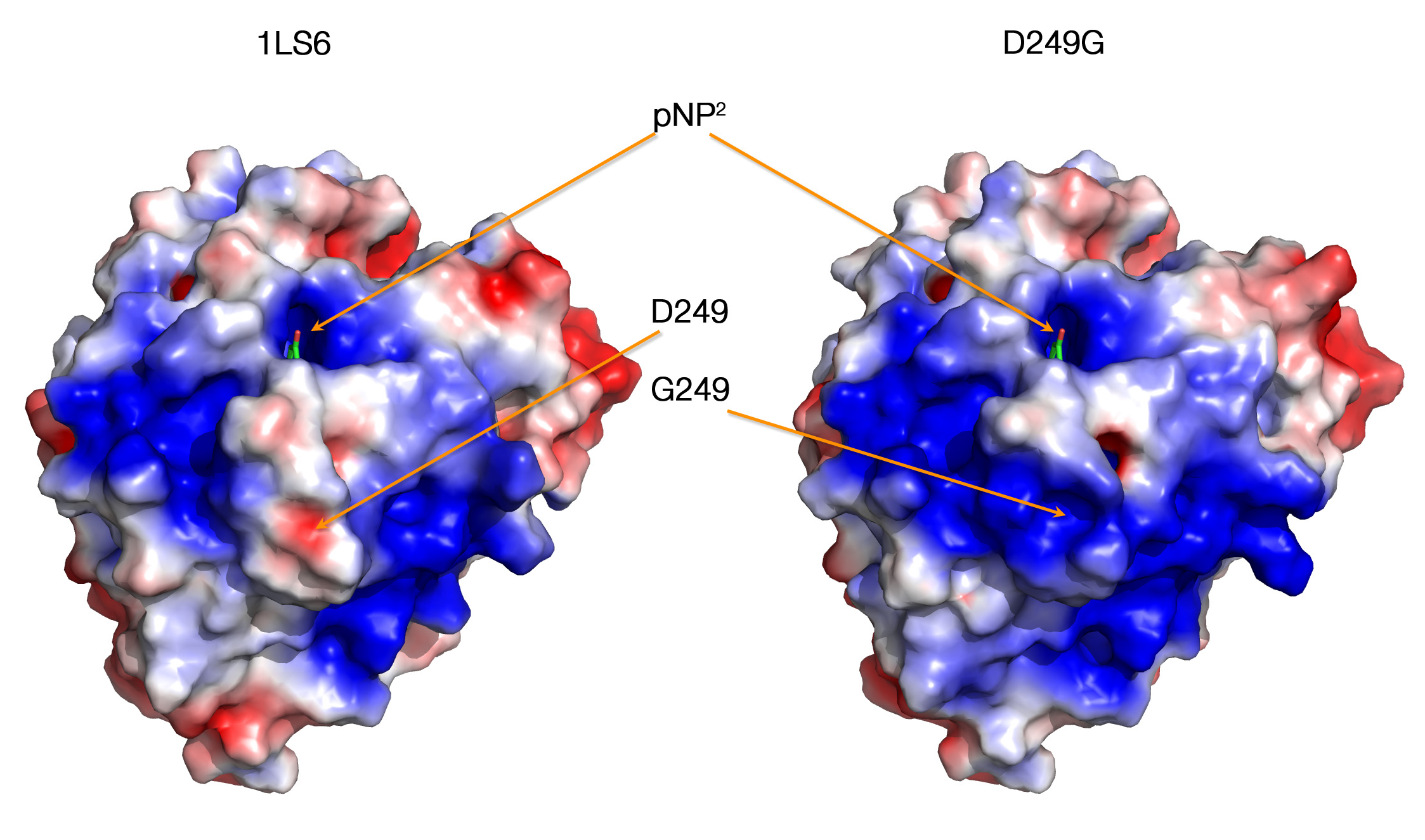

Supplement: Figure S5 — Electrostatic surface representation of SULT1A1 and SULT1A1-D249G showing a dramatic change in the surface electrostatics of the two proteins. Changes in the electrostatics of the surface can affect the pNP acceptor binding site that is located in the vicinity of Asp249. The approximate locations of Asp249 or G249 and the pNP2 molecule are highlighted by arrows. (TIF) [file pone.0026794.s005.tif]

**Table S2: Crystallization conditions for SULT1A1**

**
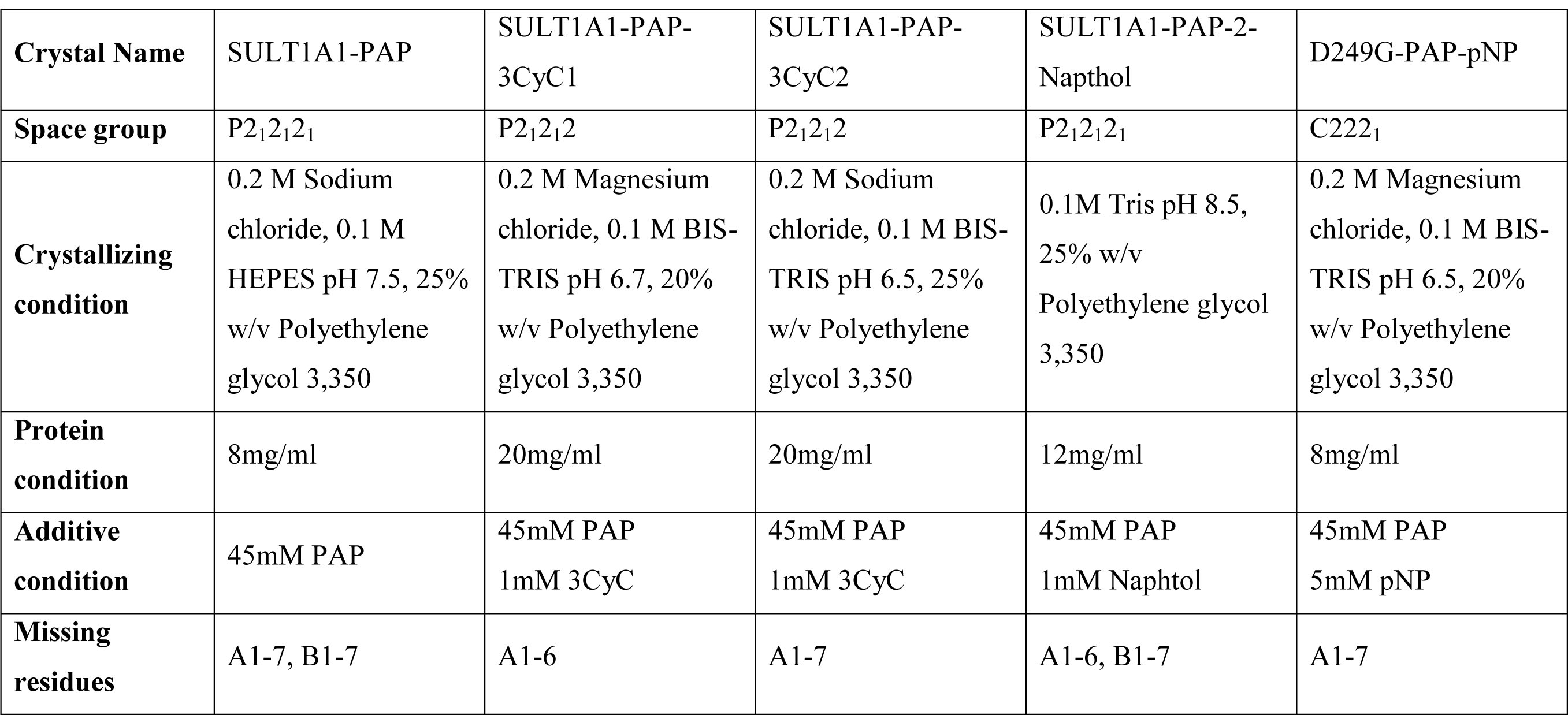
**

Supplement: Table S2 — Crystallization conditions for SULT1A1. (DOC) [file pone.0026794.s007.doc]
