# Supplementary material for: The Molecular Basis for the Broad Substrate Specificity of Human Sulfotransferase 1A1
Source: PLoS One. 2011 Nov 1;6(11):e26794. doi: 10.1371/journal.pone.0026794 (PMC3206062; doi:10.1371/journal.pone.0026794)
Supplement: Table S1 — Crystallographic statistics of SULT1A1 structures. (DOC) [file pone.0026794.s006.doc]

**Table S1. Crystallographic statistics of SULT1A1 structures**


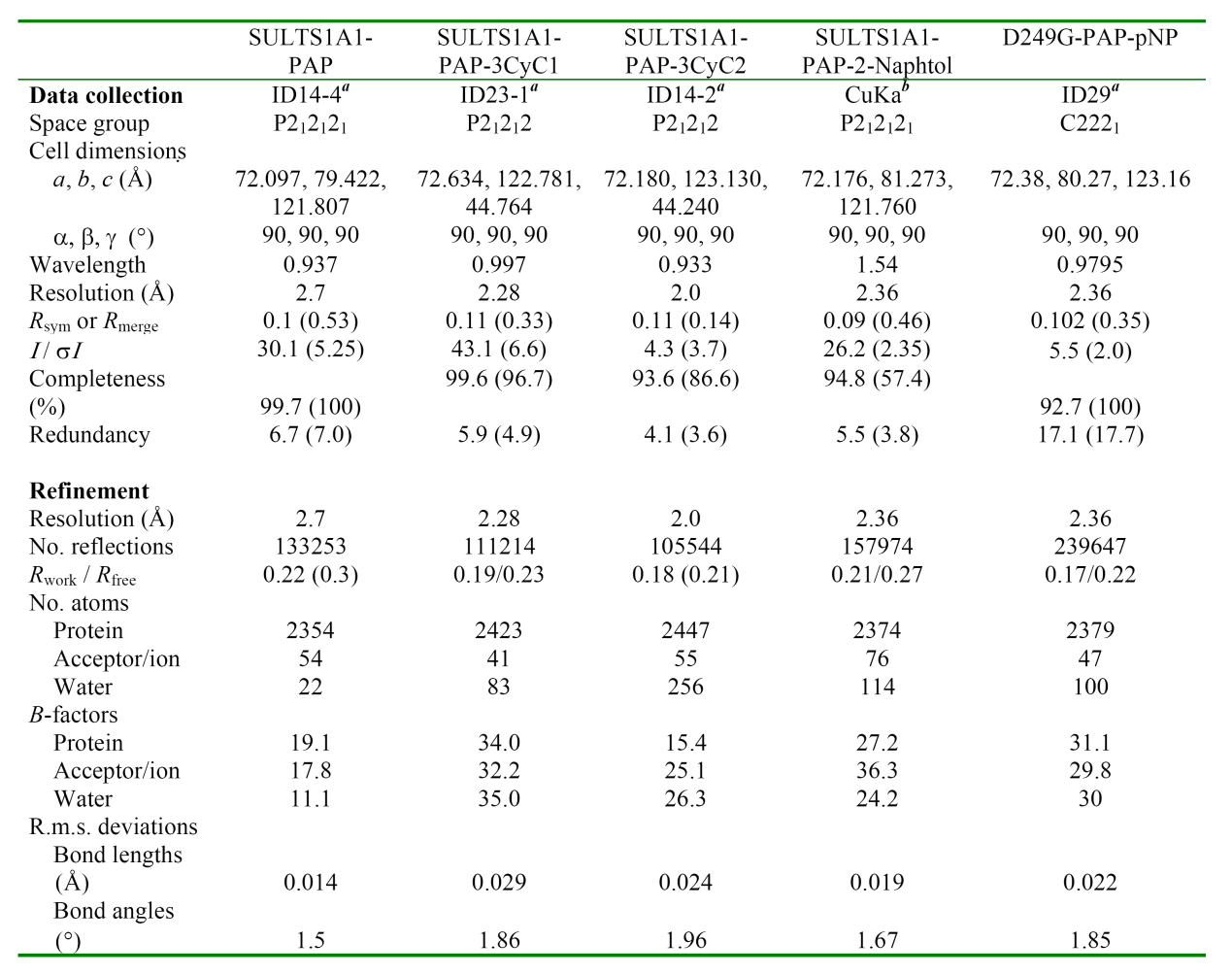


^a^These data sets were collected at the European Synchrotron Research Facility, Grenoble, France. The highest-resolution shell is shown in parentheses.

^b^This data set was collected at the Macromolecular Crystallography Research Center, Ben-Gurion University of the Negev, Be’er Sheva, Israel. The highest-resolution shell is shown in parentheses.
