# Supplementary material for: The Molecular Basis for the Broad Substrate Specificity of Human Sulfotransferase 1A1
Source: PLoS One. 2011 Nov 1;6(11):e26794. doi: 10.1371/journal.pone.0026794 (PMC3206062; doi:10.1371/journal.pone.0026794)
Supplement: Table S3 — Comparison of SULT1A1 pore size and cavity volume. (DOC) [file pone.0026794.s008.doc]

| **Table S3: Comparison of SULT1A1 pore size^a^ and cavity volume^b^** | | | | | |
| --- | --- | --- | --- | --- | --- |
|  | **No acceptor** | **2NAP** | **3CyCb** | **3CyC** | **1LS6** |
| Cavity volume (Å^3^) | 779 | 708 | 870 | 989 | 818 |
| Pore area size (Å^2^) | 45.36 | 42.84 | 51.52 | 52.40 | 53.61 |
| Pore width^c^ (Å) | 5.79 | 3.84 | 6.18 | 6.47 | 6.6 |

^a^Pore size was calculated by measuring the distance between Ile89 and Pro90, between Met77 and Phe76, and between Val243 and Pro244 and the angle between each pair of lines, after which the inputs were combined in the equation , where A is the area of the triangle, with a and b being the lines that border the angle α.

^b^Cavity volume was derived by subtracting the overall structure surface without acceptor with the surface of the whole molecule, including the acceptor. For 2NAP, the cavity could not be calculated in such a manner and was thus directly derived from the cavity calculations of the Swiss PDBviewer program.

^c^Pore width was determined by measuring the shortest distance between Phe76 and Ile89.
