# Supplementary material for: The Molecular Basis for the Broad Substrate Specificity of Human Sulfotransferase 1A1
Source: PLoS One. 2011 Nov 1;6(11):e26794. doi: 10.1371/journal.pone.0026794 (PMC3206062; doi:10.1371/journal.pone.0026794)
Supplement: Table S4 — Amino acids in human SULT1A1 that deviate from the family consensus: Comparison to SULT1A1 and SULT1 families. (DOC) [file pone.0026794.s009.doc]

**Table S4: Amino acids in human SULT1A1 that deviate from the family consensus: Comparison to SULT1A1 and SULT1 families.**

| Position^a^ | human  SULT1A1 | SULT1A1  family^b^ | SULT1 family^c^ | Mutation^d^ |
| --- | --- | --- | --- | --- |
| 56 | Q | E (6/9) | E (31/34) | Q56E |
| 67 | L | L (7/9) | V (19/34) | L67V |
| 101 | A | A (7/9) | S (22/34) | A101S |
| 117 | T | T (6/9) | S (25/34) | T117S |
| 177 | Q | Q (6/9) | K (25/34) | Q177K |
| 211 | V | V (4/9) | L (21/34) | V211L |
| 213 | H | R (7/9) | R (15/34) | H213R |
| 222 | F | L (4/9) | K (17/34) | F222K/L |
| 223 | M | I (6/9) | I (30/34) | M223I |
| 243 | V | I (5/9) | I (9/34)  L (12/34) | V243L/I |
| 247 | F | I (4/9) | I (14/34) | F247I |
| 260 | M | M (3/9) | I (15/34) | M260I |
| 266 | T | T (5/9) | N (25/34) | T266N |

^a^Amino acid residue positions are shown according to the human SULT1A1 protein sequence.

^b^The frequency of a given residue in homolog SULT1A1 proteins is based on the alignment of 9 homologous SULT1A1 proteins from different species.

^c^The frequency of a given residue in the SULT1 family is based on the alignment of 34 SULT1 proteins (including SULT1A1 homologs) from all SULT1 sub-families.

^d^Amino acid mutations spiked into hSULT1A1 to generate the ‘back-to-consensus’ library (see text for details).
