# Supplementary material for: The Molecular Basis for the Broad Substrate Specificity of Human Sulfotransferase 1A1
Source: PLoS One. 2011 Nov 1;6(11):e26794. doi: 10.1371/journal.pone.0026794 (PMC3206062; doi:10.1371/journal.pone.0026794)
Supplement: Table S5 — Mutation distribution in SULT1A1 thermostable mutants. (DOC) [file pone.0026794.s010.doc]

**Table S5: Mutation distribution in SULT1A1 thermostable mutants.**
